# Astrocyte-to-neuron interaction via NF-κB/C3/C3aR mediates chronic post-thoracotomy pain by modulating neuronal GluR1 in spinal dorsal horn

**DOI:** 10.1016/j.isci.2025.113917

**Published:** 2025-11-01

**Authors:** Wanying Mou, Ning Yu, Fengrun Sun, Huan Cui, Hanyu Zhang, Yan Cao, Sixuan Jin, Chao Ma, Afang Zhu, Lulu Ma, Yuguang Huang

**Affiliations:** 1 Department of Anesthesiology, Peking Union Medical College Hospital, Chinese Academy of Medical Sciences & Peking Union Medical College, Beijing, China; 2 State Key Laboratory of Common Mechanism Research for Major Diseases, Department of Human Anatomy, Histology and Embryology, Neuroscience Center, Institute of Basic Medical Sciences, Chinese Academy of Medical Sciences, School of Basic Medicine, Peking Union Medical College, Beijing, China; 3 Department of General Surgery, Peking Union Medical College Hospital, Peking Union Medical College & Chinese Academy of Medical Sciences, Beijing, China; 4 National Human Brain Bank for Development and Function, Beijing, China; 5 Chinese Institute for Brain Research, Beijing, China

**Keywords:** Natural sciences, Biological sciences, Neuroscience, Cell biology

## Abstract

Chronic post-thoracotomy pain (CPTP) is a debilitating postoperative complication associated with persistent hypersensitivity and neuroinflammatory changes. Here, we identify an astrocyte-neuron signaling cascade mediated by NF-κB/C3/C3aR that drives excitatory synaptic remodeling in the spinal dorsal horn during CPTP. Using a rat model, we show that the activation of astroglial NF-κB promotes C3 synthesis, which interacts with neuronal C3aR to enhance GluR1 expression and synaptic localization, thereby facilitating pain hypersensitivity. The pharmacological inhibition of NF-κB or knockdown of astroglial C3 or neuronal C3aR markedly attenuated mechanical and cold allodynia, accompanied by reduced GluR1 expression. These findings define a mechanistic link between glial NF-κB activation, complement signaling, and neuronal excitatory transmission, highlighting the NF-κB/C3/C3aR pathway as a potential therapeutic target for chronic postoperative pain.

## Highlights

• NF-κB activation in astrocytes upregulates C3 and drives CPTP progression
• Astrocyte-derived C3 activates neuronal C3aR to remodel excitatory synapses
• Targeting neuronal C3aR reduces GluR1 expression and alleviates pain behaviors
• NF-κB/C3/C3aR axis emerges as a therapeutic target for chronic post-thoracotomy pain

## Graphical abstract

**Figure:**
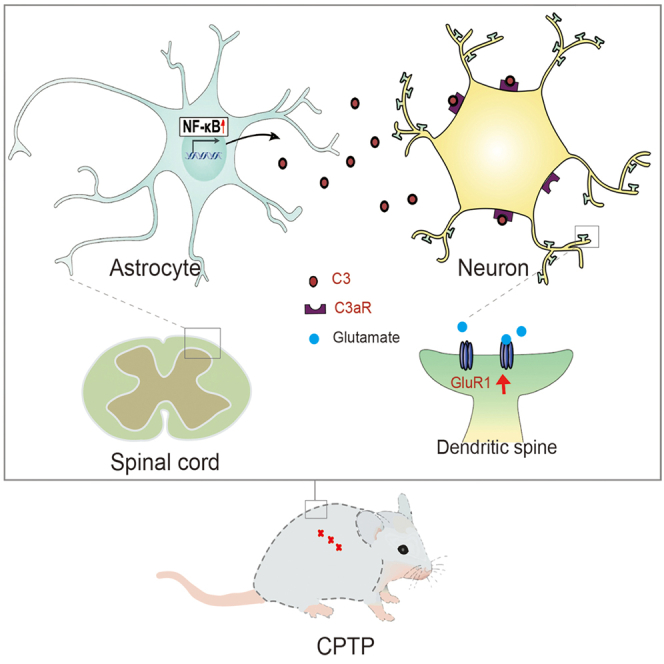

## Introduction

Chronic post-thoracotomy pain (CPTP) is a common complication following thoracotomy, with incidence rates reported to range from 20% to 82%.^1^^,^^2^^,^^3^^,^^4^^,^^5^ This persistent pain can last for months or even years after surgery, severely impairing the quality of life and increasing the risk of opioid dependence or overdose.^6^^,^^7^^,^^8^^,^^9^ Various strategies, including nerve blocks, topical anesthetics, and novel pharmacological agents, have been employed to alleviate postoperative pain and reduce opioid consumption.^4^^,^^5^^,^^7^^,^^10^ Among these, non-opioid analgesics have garnered increasing attention, particularly for their potential to prevent the transition from acute to chronic postoperative pain.

The complement system, particularly complement component 3 (C3), plays a central role in neuroinflammation.^11^ Upon activation, C3 is cleaved into two fragments: C3a and C3b. The binding of C3a to its receptor, C3aR, triggers inflammatory cascades and contributes to disease progression. Our previous work demonstrated that the activation of the spinal C3/C3aR pathway promotes the development of CPTP.^12^^,^^13^ C3aR is predominantly expressed in microglia and neurons, and its inhibition has been shown to alleviate pain by suppressing microglial C3aR-mediated M1-type inflammatory responses.^12^^,^^14^ However, the specific role of neuronal C3aR in the initiation and maintenance of CPTP remains poorly defined.

Notably, the activation of neuronal C3aR has been implicated in regulating synaptic plasticity and maintaining normal dendritic architecture.^15^ Given that α-amino-3-hydroxy-5-methyl-4-isoxazolepropionic acid receptors (AMPARs), particularly the GluR1 subunit, are central to excitatory synaptic transmission and plasticity, their roles in sensory signal processing and central sensitization have been extensively studied.^16^ Increased spinal GluR1 expression has also been observed in various models of neuropathic and inflammatory pain.^17^^,^^18^ Based on these findings, we hypothesize that neuronal C3aR in the spinal cord contributes to CPTP by modulating GluR1 expression and/or function.

In this study, we combined genetic and pharmacological approaches to investigate the nociceptive role of the NF-κB/C3/C3aR signaling axis in CPTP. In particular, we examined how this pathway regulates excitatory GluR1 expression, clarifying its contribution to the development and maintenance of CPTP.

## Results

### Chronic post-thoracotomy pain induced upregulation of astroglial C3 in the spinal dorsal horn

**Figure 1:**
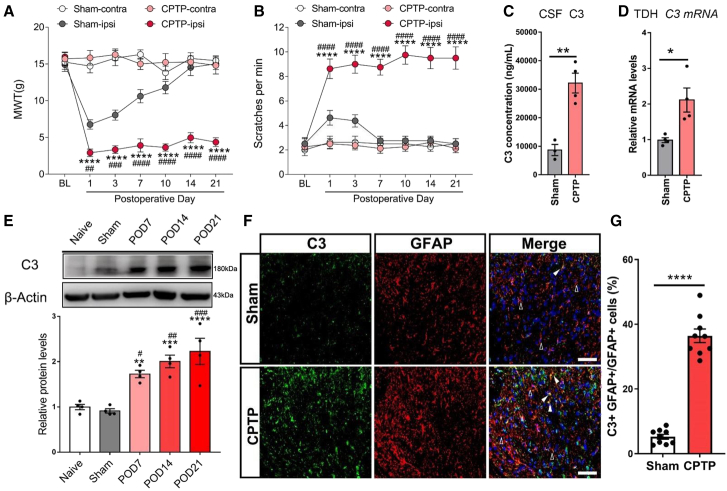
CPTP induced upregulation of astroglial C3 in the spinal dorsal horn (A) Mechanical withdrawal threshold (MWT) was measured in the ipsilateral (CPTP-ipsi) and contralateral (CPTP-contra) sides of rats compared with sham-operated controls (Sham-ipsi and Sham-contra). *n* = 6, two-way ANOVA following Bonferroni’s *post hoc* test, ∗∗∗∗*p* < 0.0001, vs. CPTP-contra; ^##^*p* < 0.01, ^###^*p* < 0.001, ^####^*p* < 0.0001, vs. Sham-ipsi. (B) Measurements of hind paw scratches and turning maneuvers challenged by cold acetone. *n* = 6, two-way ANOVA following Bonferroni’s *post hoc* test, ∗∗∗∗*p* < 0.0001, vs. CPTP-contra; ^####^*p* < 0.0001, vs. Sham-ipsi. (C) ELISA analysis of C3 concentration in the CSF from sham and CPTP rats on POD 14. *n* = 4, Student’s *t* test, ∗∗*p* < 0.01. (D) qRT-PCR analysis of C3 in the TDH from sham and CPTP rats on POD 14. *n* = 4, Student’s *t* test, ∗*p* < 0.05. (E) Western blot analysis of C3 in the TDH from naive, sham, and CPTP rats on POD 7, 14, and 21. *n* = 4, One-way ANOVA following Bonferroni’s *post hoc* test, ∗∗*p* < 0.01, ∗∗∗*p* < 0.001, ∗∗∗∗*p* < 0.0001, vs. Sham; ^#^*p* < 0.05, ^##^*p* < 0.01, ^###^*p* < 0.001, vs. Naive. (F and G) Representative images of TDH on POD 14 stained for C3 (green), GFAP (red), along with the quantitative analysis of percentage overlap. *n* = 3, Student’s *t* test, ∗∗∗∗*p* < 0.0001. Scale bars: 50 μm. See also Figures S1 and S2 for the schematic of the CPTP model and C3^+^ cell distribution in the TDH. Data are presented as mean ± SEM.

As prescribed in STAR Methods, we established a modified rat model of CPTP. Behavioral assays revealed a significant reduction in MWT (Figure 1A) and an increase in acetone-induced scratches (Figure 1B) on the ipsilateral (surgical) side in the CPTP group from POD 1 to 21, compared with the contralateral side and the sham controls. Unlike the classical model, in which only approximately 50% of animals develop CPTP, nearly all rats subjected to modified thoracotomy exhibited CPTP. Furthermore, both protein levels of C3 in the CSF, tested by ELISA, and its mRNA levels in the T4-T5 TDH were upregulated in CPTP rats on POD 14 (Figures 1C and 1D). In addition, a progressive increase in C3 protein expression was observed in the T4-T5 TDH from POD 7 to POD 21 (Figure 1E). Regarding cellular localization, immunofluorescence analysis revealed the colocalization of C3 with glial fibrillary acidic protein (GFAP, an astrocyte marker) in CPTP rats compared with sham-operated controls on POD 14, suggesting that astroglial C3 expression was increased in CPTP rats (Figures 1F and 1G). Although a minor subset of C3^+^ cells did not colocalize with GFAP, the double-immunofluorescence results (CPTP POD 14) showed that C3^+^ cells were predominantly GFAP^+^ astrocytes, with little to no colocalization with IBA1^+^ microglia or NeuN^+^ neurons. (Figure S2). Collectively, these findings indicate that spinal C3 expression, particularly in astrocytes, is markedly elevated in response to CPTP.

### Astroglial p-NF-κB mediated C3 synthesis

**Figure 2:**
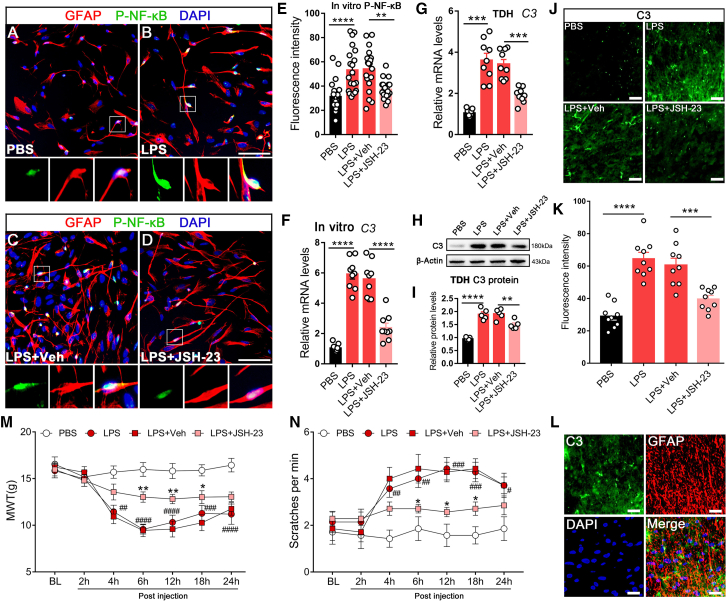
Astroglial p-NF-κB mediated C3 synthesis *in vitro* and *in vivo* (A and B) Representative images of primary cultured astrocytes treated with PBS or LPS and stained for GFAP (red) and p-NF-κB (green). Scale bars: 100 μm. Images below each panel show enlarged views of the bracketed areas. (C and D) Representative images of LPS-stimulated primary astrocytes treated with vehicle or JSH-23. Scale bars: 100 μm. Images below each panel show enlarged views of the bracketed areas. (E) Quantification of the fluorescence intensity of p-NF-κB between treatment groups. Student’s *t* test, ∗∗*p* < 0.01, ∗∗∗∗*p* < 0.0001. (F) mRNA expression of C3 in primary astrocytes among different groups. Student’s *t* test, ∗∗∗∗*p* < 0.0001. (G) mRNA expression of C3 in the TDH from rats administered with PBS, LPS, LPS + Vehicle, and LPS + JSH-23 on POD 14. *n* = 3, Student’s *t* test, ∗∗∗*p* < 0.001. (H and I) Representative gel bands of C3 and p-NF-κB in the TDH from rats with different treatments, along with the quantitative analysis of C3 protein. *n* = 3, Student’s *t* test, ∗∗*p* < 0.01, ∗∗∗∗*p* < 0.0001. (J and K) Representative images of TDH from rats injected with multiple drugs stained for C3, along with the quantification of the fluorescence intensity of C3. *n* = 3, Student’s *t* test, ∗∗∗*p* < 0.001, ∗∗∗∗*p* < 0.0001. Scale bars: 50 μm. (L) Representative images of immunofluorescence staining for C3 and GFAP in the TDH from rats treated with LPS on POD 14. Scale bars: 50 μm. (M) MWT in the thoracic surgical region of rats with multiple treatments. *n* = 6, two-way ANOVA following Bonferroni’s *post hoc* test, ∗*p* < 0.05, ∗∗*p* < 0.01, vs. LPS + Veh; ^##^*p* < 0.01, ^###^*p* < 0.001, ^####^*p* < 0.0001, vs. PBS. (N) Measurements of hind paw scratches and turning maneuvers challenged by cold acetone. *n* = 6, two-way ANOVA following Bonferroni’s *post hoc* test, ∗*p* < 0.05, vs. LPS + Veh; ^##^*p* < 0.01, ^###^*p* < 0.001, vs. PBS. Data are presneted as mean ± SEM.

Given the significance of aberrant NF-κB activation in neuroinflammatory conditions and the potential role of the regulation of NF-κB in C3 formation, we further investigated the involvement of NF-κB in C3 synthesis and its contribution to CPTP pathogenesis. LPS is commonly used to induce p-NF-κB activation both *in vivo* and *in vitro*. Initially, primary astrocytes were treated with LPS to validate the effect of p-NF-κB on C3 activation. Compared with the PBS-treated group, LPS stimulation significantly increased p-NF-κB expression (Figures 2A and 2B), which was effectively suppressed by the administration of the p-NF-κB inhibitor JSH-23 (Figures 2C–2E). Subsequent qRT-PCR analysis revealed a marked increase in C3 mRNA expression following LPS treatment, which was significantly inhibited by JSH-23 administration (Figure 2F). The data above suggest that p-NF-κB is involved in the synthesis of astroglial C3 *in vitro*.

To validate these findings *in vivo*, further experiments were conducted on naive rats. LPS or PBS was administered via intrathecal injection. Subsequently, LPS-treated rats received intrathecal injections of either JSH-23 or vehicle control. Western blot and qRT-PCR analyses revealed increased C3 expression in the TDH of LPS-treated rats, which was further inhibited by JSH-23 (Figures 2G–2I). Immunofluorescence staining of TDH further supported the notion that LPS induced C3 expression in the TDH, which was attenuated by JSH-23 (Figures 2J and 2K). Subsequently, immunofluorescence staining suggested that the elevated C3 induced by LPS was mainly colocalized with astrocytes in the TDH (Figure 2L). Moreover, LPS-induced mechanical and cold allodynia were significantly attenuated by inhibiting p-NF-κB through JSH-23 administration (Figures 2M and 2N).

### p-NF-κB inhibition alleviated chronic post-thoracotomy pain and downregulated C3

**Figure 3:**
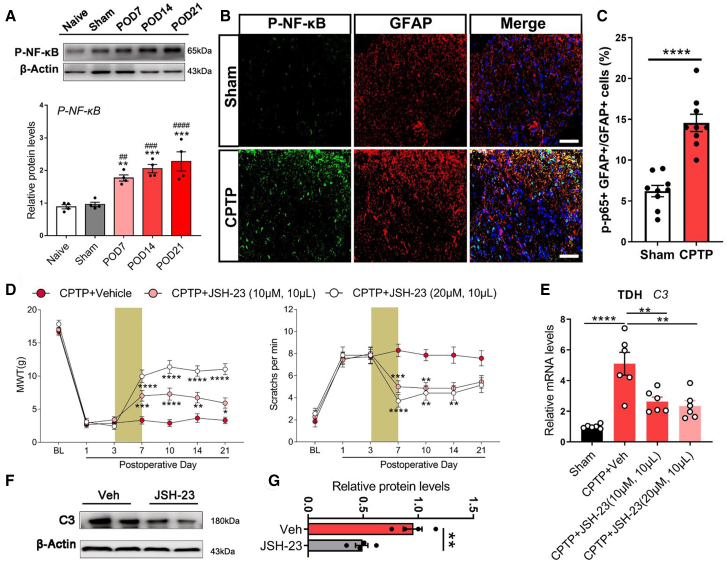
p-NF-κB inhibition alleviated CPTP and downregulated C3 (A) Western blot analysis of p-NF-κB in the TDH from naive, sham, and CPTP rats on POD7, 14, and 21. One-way ANOVA following Bonferroni’s post hoc test, ∗∗*p* < 0.01, ∗∗∗*p* < 0.001, vs. Sham; ##*p* < 0.01, ###*p* < 0.001, ####*p* < 0.0001, vs. Naive. (B and C) Immunofluorescence staining of p-NF-κB (green) and GFAP (red) in the TDH from sham and CPTP rats on POD 14, along with the quantification of percentage overlap. *n* = 3, Student’s *t* test, ∗∗∗∗*p* < 0.0001. Scale bars: 50 μm. (D) MWT and scratches in CPTP rats treated with vehicle or NF-κB inhibitor JSH-23. The brown rectangle was indicated as drug administration. *n* = 6, two-way ANOVA following Bonferroni’s post hoc test, ∗*p* < 0.05, ∗∗*p* < 0.01, ∗∗∗*p* < 0.001, ∗∗∗∗*p* < 0.0001. (E) qRT-PCR analysis of C3 mRNA in the TDH from rats with different treatments. One-way ANOVA following Bonferroni’s post hoc test, ∗∗*p* < 0.01, ∗∗∗∗*p* < 0.0001. (F-G) Western blot analysis of C3 in the TDH from CPTP rats injected with vehicle or JSH-23 (20 μM) on POD 14. *n* = 3, Student’s *t* test, ∗∗*p* < 0.01. See also Figure S3, which shows the identification of the inhibitory effects of JSH-23 on NF-κB, related to this figure. Data are presented as mean ± SEM.

Given that astroglial p-NF-κB mediates C3 synthesis both *in vivo* and *in vitro*, we further investigated its regulatory role in the development of CPTP. Notably, p-NF-κB expression in the TDH was elevated from POD 7 to 21 following CPTP induction (Figure 3A). Enhanced colocalization of p-NF-κB and GFAP was also observed in the CPTP group (Figures 3B and 3C), consistent with the upregulation of astroglial p-NF-κB in the TDH. To further investigate the functional role of p-NF-κB in CPTP, JSH-23 was administered to CPTP rats. The behavioral testing revealed that the MWT was increased, and acetone-challenged scratches were decreased when exposed to different concentrations of JSH-23 treatment (Figure 3D). The Western blot test confirmed the inhibitory effects of JSH-23 on NF-κB (Figure S3). Moreover, both qRT-PCR analysis and Western blot analyses demonstrated a reduction of C3 expression in CPTP rats after inhibiting p-NF-κB (Figures 3E–3G). These findings suggest that astroglial p-NF-κB activation promotes C3 synthesis, and its inhibition may, at least in part, alleviate CPTP by suppressing C3 expression.

### C3 induced nociceptive effects and synaptic changes via C3aR

**Figure 4:**
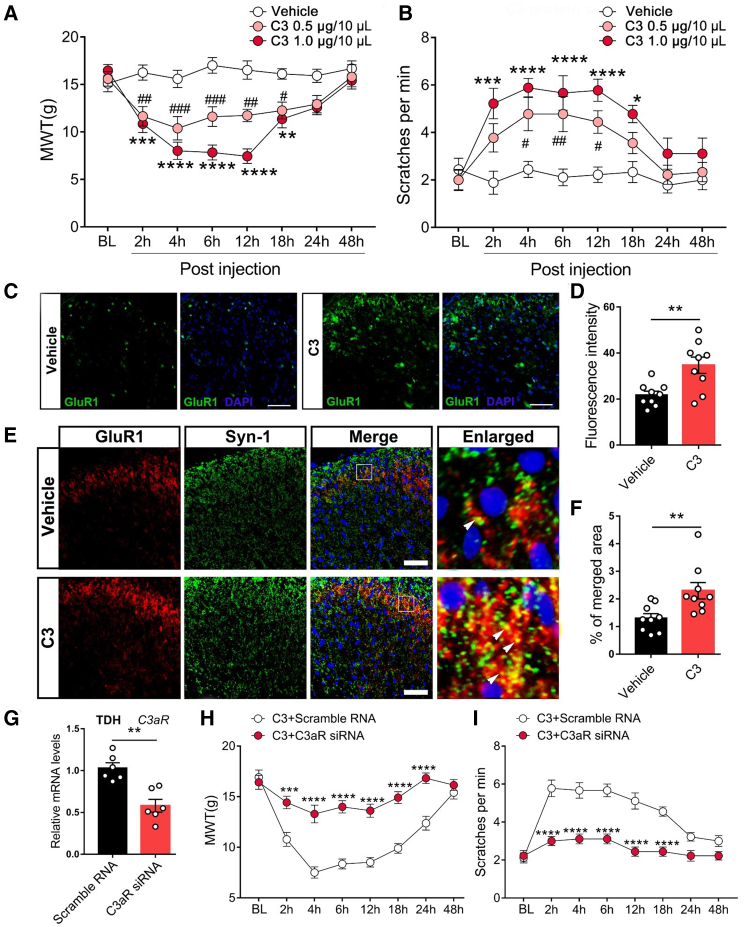
C3 induced nociceptive effects and synaptic changes via C3aR (A) MWT in the thoracic surgical region of rats treated with C3 or vehicle. *n* = 6, two-way ANOVA following Bonferroni’s *post hoc* test, ∗*p* < 0.05, ∗∗*p* < 0.01, ∗∗∗*p* < 0.001, ∗∗∗∗*p* < 0.0001, vs. Vehicle. (B) Measurements of hind paw scratches and turning maneuvers challenged by cold acetone. *n* = 6, two-way ANOVA following Bonferroni’s *post hoc* test, ∗*p* < 0.05, ∗∗∗*p* < 0.001, ∗∗∗∗*p* < 0.0001, vs. Vehicle; ^#^*p* < 0.05, ^##^*p* < 0.01, vs. Vehicle. (C and D) Representative images of TDH from rats treated with vehicle or C3 (1.0 μg) stained for GluR1 on POD 14, along with the quantitative analysis. *n* = 3, Student’s *t* test, ∗∗*p* < 0.01. Scale bars: 50 μm. (E and F) Representative images of GluR1 and Syn-1 immunostaining in the TDH from rats injected with C3 and vehicle, along with the quantitative analysis of the percentage of overlap. Images on the right are an enlarged view of bracketed areas on POD 14. *n* = 3, Student’s *t* test, ∗∗*p* < 0.01. Scale bars: 50 μm. (G) qRT-PCR analysis of C3aR in the TDH from C3-treated rats transfected by scramble RNA or C3aR siRNA. *n* = 3, Student’s *t* test, ∗∗*p* < 0.01. (H and I) MWT and scratches of C3-treated rats transfected by scramble RNA or C3aR siRNA. *n* = 6, two-way ANOVA following Bonferroni’s *post hoc* test, ∗∗∗*p* < 0.001, ∗∗∗∗*p* < 0.0001. Data are presented as mean ± SEM.

To further investigate the nociceptive effects of C3, we evaluated behavioral outcomes following intrathecal injection (*i.t.*) of C3 (0.5 or 1 μg/10 μL) in naive rats. C3 injection led to a rapid decrease in the MWT and triggered increased scratching behavior in response to acetone stimulation (Figures 4A and 4B). Additionally, both mechanical and cold allodynia induced by C3 persisted for up to 18 h. We next investigated the impact of C3 on AMPA glutamate receptors. Immunofluorescence staining revealed a significant upregulation of the GluR1 subunit of AMPA receptor after C3 administration, compared with the vehicle-treated controls (Figures 4C and 4D). Moreover, the colocalization of GluR1 with the synaptic marker Synapsin 1 (Syn-1) was also increased in C3-treated rats, consistent with a C3-associated enhancement of excitatory synaptic remodeling (Figures 4C and 4D). We further assessed whether the downstream receptor C3aR mediated the nociceptive effects of C3. siRNA targeting C3aR was administered, and its knockdown efficiency in the TDH was confirmed by qRT-PCR (Figure 2G). Notably, our results showed that C3aR siRNA, but not the scramble RNA, significantly alleviated C3-induced mechanical and cold allodynia (Figures 4H and 4I). These findings support the notion that C3 exerts nociceptive effects through a C3aR-dependent mechanism and promotes AMPA receptor-associated synaptic remodeling.

### GluR1 was regulated by C3 suppression under chronic post-thoracotomy pain conditions

**Figure 5:**
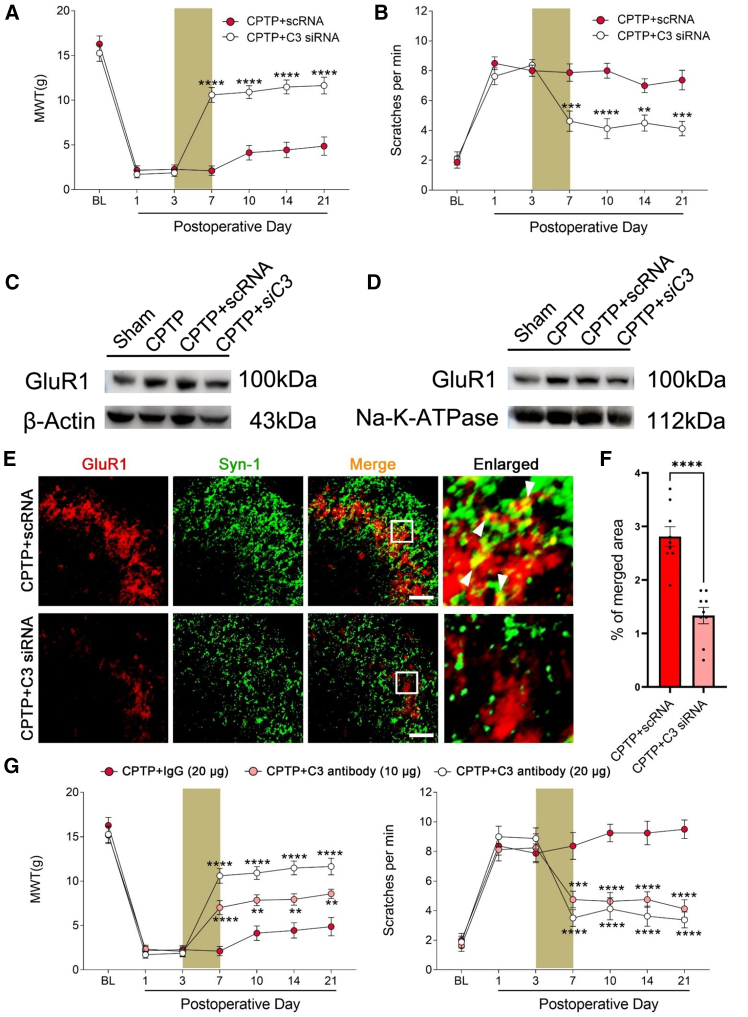
GluR1 was regulated by C3 suppression under CPTP conditions (A and B) MWT and scratches in the thoracic surgical region of CPTP rats transfected by scramble RNA (scRNA) or C3 siRNA. The brown rectangle was indicated as siRNA administration. *n* = 6, two-way ANOVA following Bonferroni’s *post hoc* test, ∗∗*p* < 0.01, ∗∗∗*p* < 0.001, ∗∗∗∗*p* < 0.0001. (C and D) Western blot analysis of whole cell GluR1 (C) and membranal GluR1 (D) among Sham, CPTP, CPTP+scRNA, and CPTP+siC3 groups on POD 14. (E and F) Representative images of GluR1 (red) and Syn-1 (green) in the TDH from CPTP rats transfected with scRNA or C3 siRNA on POD 14, along with the quantification of the percentage of overlap. Images on the right are enlarged views of bracketed areas. Scale bars: 50 μm. (G) MWT and scratches of CPTP rats treated with isotype IgG or C3 antibody. The brown rectangle was indicated as C3 antibody administration. *n* = 6, two-way ANOVA following Bonferroni’s *post hoc* test, ∗∗*p* < 0.01, ∗∗∗*p* < 0.001, ∗∗∗∗*p* < 0.0001, vs. CPTP+IgG. See also Figure S4 for the effect of siC3 on GluR1 expression in the TDH of the CPTP model, related to this figure. Data are presented by mean ± SEM.

As previously demonstrated, C3 exerts nociceptive effects in naive rats, which are associated with GluR1 upregulation and C3aR activation. We further investigated their role and regulation in the context of CPTP. Intrathecal administration of C3 siRNA (siC3) was employed to suppress C3 synthesis from POD 3 to 7. Western blot analysis confirmed that siC3 significantly downregulated the C3 protein expression in the TDH (Figures S4A and S4B). Notably, rats treated with C3 siRNA exhibited a significant elevation in MWT compared with those treated with scramble RNA (Figure 5A). Furthermore, the frequency of acetone-induced scratches was reduced in the C3 siRNA group (Figure 5B). In addition, GluR1 mRNA expression in the TDH was markedly decreased following C3 knockdown (Figure S4C). For the protein levels, both were significantly downregulated by siC3 (Figures 5C–5D, Figures S4D, and S4E). Given our previous observation of C3-induced enhancement in the colocalization of GluR1 and Syn-1, we performed immunofluorescence staining to assess the impact of C3 suppression on this colocalization. Remarkably, we detected a reduction in the colocalization of GluR1 and Syn-1 when C3 synthesis was inhibited (Figures 5E and 5F). Consistently, the alleviation of both mechanical and cold allodynia in CPTP rats was also observed when neutralizing C3 by intrathecally injecting C3 antibody from POD 3 to 7 (Figure 5G). These findings provided further evidence that C3 contributes to CPTP pathogenesis by regulating GluR1 expression.

### Neuronal C3aR knockdown attenuated chronic post-thoracotomy pain and decreased GluR1 expression

**Figure 6:**
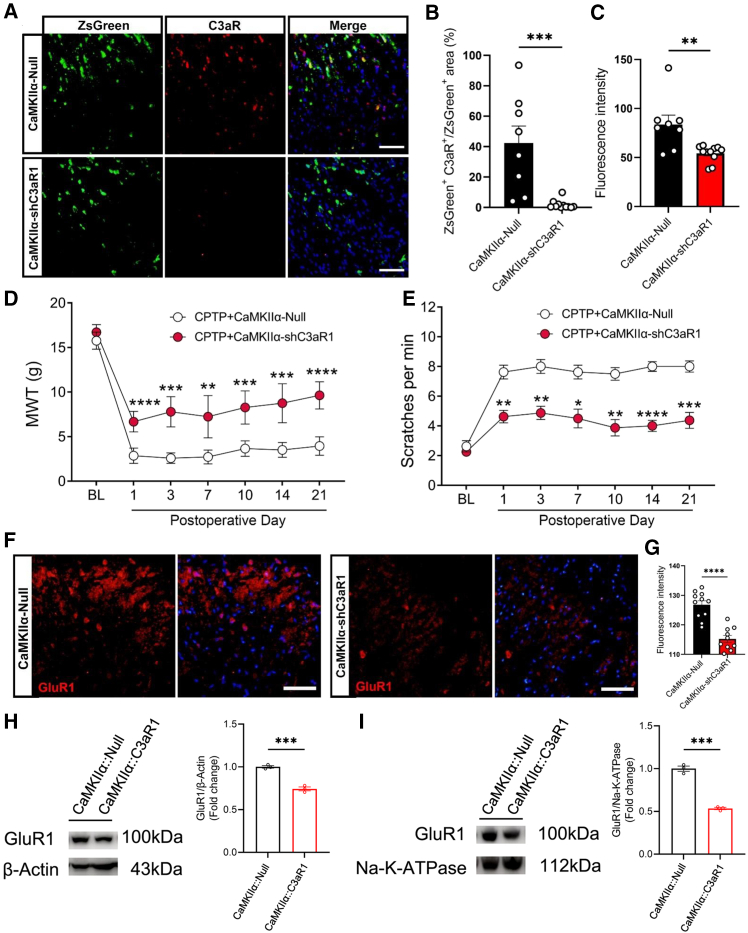
Neuronal C3aR knockdown attenuated CPTP and was associated with decreased GluR1 expression (A and C) Representative images of ZsGreen (green) and C3aR (red) in the TDH from rats treated with AAV, along with the quantitative analysis of colocalization area and C3aR fluorescence intensity. *n* = 3, Student’s *t* test, ∗∗*p* < 0.01, ∗∗∗*p* < 0.001. Scale bars: 50 μm. (D) MWT in the thoracic surgical region of rats treated with AAV. *n* = 6, two-way ANOVA following Bonferroni’s *post hoc* test, ∗∗*p* < 0.01, ∗∗∗*p* < 0.001, ∗∗∗∗*p* < 0.0001. (E) Measurements of hind paw scratches and turning maneuvers challenged by cold acetone. *n* = 6, two-way ANOVA following Bonferroni’s *post hoc* test, ∗*p* < 0.05, ∗∗*p* < 0.01, ∗∗∗*p* < 0.001, ∗∗∗∗*p* < 0.0001. (F and G) Representative images of GluR1 in the TDH from CPTP rats injected with AAV, along with the quantification of fluorescence intensity. *n* = 3, Student’s *t* test, ∗∗∗∗*p* < 0.0001. Scale bars: 50 μm. (H) Representative images of GluR1 and β-Actin among Null and CaMKII::C3aR1 groups. *n* = 3, *t* test, ∗∗∗*p < 0.001*. (I) Representative images of GluR1 and Na-K-ATPase among the Null and CaMKII::C3aR1 groups. *n* = 3, Student’s *t* test, ∗∗∗*p < 0.001*. See also Figure S5, related to this figure, showing the mRNA expression of GluR1 in the TDH between the Null and CaMKII::C3aR1 groups. Data are presented by mean ± SEM.

Based on our previous findings showing the notable expression of C3aR in both neurons and microglia, along with the established pronociceptive role of microglial C3aR in CPTP,^14^ we further investigated the regulatory mechanisms linking neuronal C3aR to CPTP. To achieve C3aR knockdown, we employed the intrathecal administration of adeno-associated virus (AAV, specifically, AAV9-CaMKIIα-zsgreen-shC3aR and AAV9-CaMKIIα-zsgreen-Null, sourced from HanBio). Immunofluorescence staining of the spinal cord was performed to assess the efficiency of C3aR knockdown. Notably, the colocalization area fraction of ZsGreen and C3aR, as well as the fluorescence intensity of C3aR, exhibited a significant reduction following AAV-shC3aR injection, indicating the successful knockdown of C3aR in excitatory interneurons (Figures 6A–6C). Behavioral testing revealed a notable reduction in both mechanical and cold allodynia from POD 1 to 21 (Figures 6D and 6E). Furthermore, GluR1 expression in the TDH was significantly decreased at POD 14 following AAV-mediated C3aR knockdown (Figures 6F and 6G). We further tested the effects of neural specific C3aR knockdown on GluR1 expression. The GluR1 mRNA was significantly downregulated by neuronal C3aR knockdown (Figure S5). Furthermore, the Western blot test showed that neuronal C3aR knockdown significantly downregulated both total cell GluR1 expression (Figure 6H) and membranal GluR1 expression (Figure 6I). Collectively, our findings suggest that C3aR in excitatory interneurons contributes to CPTP and may contribute to pronociceptive effects, at least in part, through the modulation of GluR1 expression.

## Discussion

### Main findings and model optimization

Our study demonstrates that spinal astroglial NF-κB promotes C3 synthesis, which contributes to the development of CPTP through interaction with neuronal C3aR. Importantly, the inhibition of the NF-κB/C3/C3aR signaling axis effectively attenuated GluR1 upregulation and excitatory synaptic transmission of GluR1.

CPTP affects up to 82% of patients following thoracotomy.^3^^,^^4^^,^^5^ Despite clinical efforts, including multiple trials to reduce postoperative pain, analgesic efficacy remains inconsistent and inconclusive.^19^^,^^20^^,^^21^^,^^22^ Here, we employed a murine model to elucidate the molecular mechanisms underlying CPTP and to identify novel therapeutic targets. Notably, only about half of the rats subjected to thoracotomy developed persistent pain behaviors, reflecting both increased variability and consistency in clinical morbidity rates. Several risk factors have been implicated in the high occurrence of CPTP, including the degree and duration of rib retraction and surgical tissue-handling technique.^21^^,^^23^ Indeed, a previous study reported a positive correlation between prolonged retraction and chronic pain incidence.^24^ To address this, we optimized the CPTP model by extending rib retraction from 60 min to 3 h, thereby achieving a more reliable induction of chronic postoperative pain. Consistently, our results showed that most rats exhibited mechanical hyperalgesia and cold allodynia from POD 1, persisting for over three weeks, confirming the successful establishment of a stable and reproducible CPTP model.

### C3/C3aR signaling and synaptic remodeling

As the central component of the complement cascade, C3 is dysregulated in multiple neurological diseases, particularly neurodegenerative diseases.^25^^,^^26^^,^^27^^,^^28^ Previous studies have demonstrated that enhanced C3/C3aR signaling promotes synaptic loss in models of Alzheimer’s disease and multiple sclerosis,^18^ whereas C3 deficiency confers neuroprotection against cognitive decline,^29^ and hippocampal CA3 synapse and neuron loss.^28^^,^^30^^,^^31^ In the present study, our results suggest that the astroglial C3 interacts with neuronal C3aR to drive excitatory synaptic remodeling in the spinal cord by upregulating and facilitating synaptic trafficking of GluR1. Conversely, knockdown of the C3/C3aR axis attenuated synaptic remodeling and thoracotomy-induced pain behaviors. Consistent with us, the complement-dependent regulation of GluR1 has also been reported in murine models of lupus cerebritis.^32^ Interestingly, C3aR appears to exert cell type specific effects. Microglial C3aR has been implicated in synaptic engulfment,^33^^,^^34^ whereas neuronal C3aR contributes to synaptic excitation in CPTP rats. This divergence may reflect the activation of distinct downstream signaling cascades in different cellular contexts, whereby neuronal C3aR preferentially promotes synaptogenesis while microglial C3aR facilitates synaptic elimination. Further investigation into astroglial C3 activity in CPTP will be critical to declinate the broader contribution of C3/C3aR signaling to chronic pain pathogenesis. More broadly, both synaptic loss in neurodegenerative diseases and increased excitatory synapses in chronic pain may represent adaptive responses to pathological changes in the local microenvironments and inflammatory milieu.

### CaMKIIα and GluR1 as downstream effectors

The superficial spinal dorsal horn (SDH), densely innervated by nociceptive primary afferents, serves as the first site of the synaptic integration of pain signals and contains a high density of excitatory glutamatergic interneurons. CaMKII is a Ca^2+^-dependent kinase with diverse cellular functions, among which the CaMKIIα isoform is the most abundant protein at excitatory synapses and exhibits selectivity for glutamatergic neurons.^35^ Accordingly, the CaMKIIα promoter has been widely employed to selectively target excitatory neurons in the forebrain.^36^ A recent study demonstrated that the inhibition of CaMKIIα-positive neurons in layer II/III of the medial prefrontal cortex alleviates paclitaxel-evoked pain behaviors in mice.^37^ Moreover, CaMKII phosphorylation has been shown to suppress retinal injury and protect long-distance retinal ganglion cell axon projection.^38^ In addition, CaMKII stabilizes synaptic AMPARs indirectly through interactions with postsynaptic proteins and the actin cytoskeleton, thereby supporting synaptic repair and structural stabilization.^39^^,^^40^ Collectively, these findings suggest that CaMKII plays a critical role in synaptic regulation and plasticity. Building on these insights, our results demonstrate that selective knockdown of C3aR in CaMKII-positive excitatory neurons of the SDH, using AAV9-CaMKIIα-ZsGreen-shC3aR, not only reduced GluR1 expression but also alleviated mechanical and cold allodynia in CPTP rats. Given that GluR1 enhances synaptic transmission and neuronal excitability, while its downregulation contributes to pain relief,^41^^,^^42^ we propose that C3aR may act as an upstream regulator of CaMKII-GluR1 signaling. This signaling cascade may represent a core mechanism underlying CPTP-related pain and warrant further investigation as a potential therapeutic target.

### NF-κb as a central regulator of astrocytic C3

Reciprocal interaction between neurons and glial cells is increasingly recognized as fundamental mechanisms underlying the pathophysiology of chronic pain. In the spinal cord, microglia are among the first responders, activated by neuron-derived mediators such as adenosine triphosphate and calcitonin gene-related peptide.^43^^,^^44^ Once activated, microglia adopt a pro-inflammatory phenotype and release chemokines and cytokines that directly modulate excitatory synaptic transmission by enhancing glutamate release and increasing the surface expression of AMPARs. Accumulating evidence further suggests that astrocytes also secrete signaling molecules and contribute to excitatory synapse formation via AMPAR signaling.^45^ Consistent with this, our findings demonstrate that astrocytes enhance the expression of the GluR1 subunit of AMPARs through the release of C3, thereby facilitating ascending pain signaling.

We investigated the upstream regulatory signals controlling the C3/C3aR axis. NF-κB, a pivotal transcription factor, regulates the expression of numerous pro-inflammatory genes and plays a central role in neuroinflammation and chronic pain.^46^ Although NF-κB is present in multiple cell types, its role in pain modulation is predominantly linked to astrocytic activation. Astrocytic NF-κB upregulation has been reported in models of Complete Freund’s adjuvant-induced inflammation, nerve injury-induced pain, and CPTP.^47^^,^^48^^,^^49^ Clinically, increased NF-κB has been detected in peripheral blood mononuclear cells of patients with thoracotomy postoperatively compared to preoperative levels,^50^ and elevated NF-κB p65 has been observed in the dorsal root ganglia of thoracotomy-induced pain models.^49^ Importantly, the inhibition of astrocytic NF-κB signaling alleviates pain-related behaviors.

In our study, although only ∼15% of GFAP+ astrocytes were p-p65^+^ at POD 14, this proportion still represented a more than 2-fold increase compared with sham animals, suggesting that even partial astrocytic activation may exert significant functional consequences in neuron-glia communication. We further hypothesize that NF-κB activation in astrocytes follows a transient, “radiofrequency-like” pattern, rather than being continuously sustained. This notion is supported by our finding that only ∼40% of GFAP^+^ astrocytes were C3^+^ at POD 7. Since immunofluorescence essentially provides a single time-point “snapshot,” these modest proportions are better interpreted as reflecting dynamic and temporally restricted activation windows rather than cumulative engagement. In this way, transient NF-κB/C3 activation may initiate a self-perpetuating inflammatory cascade that drives long-lasting pain behaviors, thereby explaining how short-lived interventions targeting this pathway can yield persistent therapeutic effects.

Building on *in vitro* evidence that the deletion of an NF-κB inhibitor increases C3 expression,^51^ our results confirm the NF-κB-mediated transcriptional regulation of C3 in astrocytes in our modified CPTP model. Through this mechanism, astrocytic NF-κB facilitates neuron-glia communication by promoting C3 release and neuronal C3aR activation, thereby contributing to pain chronification.

### Limitations of the study

Despite the promising findings of this study, several limitations should be acknowledged. First, C3 expression was only accessed at POD 7, whereas nociceptive hypersensitivity emerged as early as POD 1. This time point was chosen to distinguish chronic pain mechanisms from acute postoperative responses; however, evaluating earlier stages in future studies would help clarify the temporal relationship between C3 activation and pain onset. Second, although astrocytes constituted the majority of C3^+^ cells in CPTP, as shown by comparative analysis of C3^+^GFAP^+^, C3^+^NeuN^+^, and C3^+^IBA1^+^ populations (Figure S2), only ∼40% of GFAP^+^ astrocytes were C3^+^ at POD 7, and only ∼15% were p-p65^+^ at POD 14. These relatively modest proportions suggest that NF-κB activation and C3 induction in astrocytes are partial and temporally heterogeneous rather than uniform. A larger proportion of astrocytes may undergo transient activation at different stages throughout CPTP, but this remains unresolved due to current technical limitations. Third, other neural cell populations, particularly neurons and microglia in deeper dorsal horn laminae, may also contribute to NF-κB activation. This possibility warrants further investigation using cell type-specific or inducible approaches. Fourth, *in vitro* co-localization of C3 and GFAP was confirmed only in the LPS-treated group, where C3 expression was sufficiently robust for reliable staining; under other conditions, weak C3 signals precluded accurate quantification. Fifth, while the AAV-CaMKIIα-shC3aR strategy enabled stable neuronal knockdown, it lacked temporal control, limiting the exploration of dynamic astrocyte-neuron interactions. Finally, although the transient inhibition of NF-κB/C3 signaling between POD 3–7 yielded long-lasting behavioral improvements, these effects are more likely attributable to the disruption of a self-perpetuating inflammatory cascade than to persistent activity of siRNA or antibody treatment.

## Resource availability

### Lead contact

Requests for further information and resources should be directed to and will be fulfilled by the lead contact, Afang Zhu (woshiafang@yeah.net).

### Materials availability

There are no additional data, software, databases, or applications/tools available beyond those disclosed in the current study. All data are included in the article and supplementary data section.

### Data and code availability

• This article does not report original datasets.
• This article does not report original code.
• Any additional information required to reanalyze the data reported in this article is available from the lead contact upon request.

## Acknowledgments

This study was supported by grants from the 10.13039/501100001809National Natural Science Foundation of China (#82071252) and the 10.13039/501100001809National Natural Science Foundation of China (#81901148).

## Author contributions

Yuguang Huang provided the original idea and platform support. Afang Zhu designed the project. Yuguang Huang, Chao Ma, and Lulu Ma provided invaluable guidance throughout the experiments. Wanying Mou, Hanyu Zhang, and Ning Yu contributed to executing animal models, behavioral tests, drug/virus administration, and tissue collection. Immunofluorescence staining, Western blotting, ELISA, and qRT-PCR experiments were conducted by Wanying Mou, Ning Yu, and Fengrun Sun. Yan Cao and Sixuan Jin assisted with immunofluorescence staining and Western blot image analysis. Data analysis and article writing were carried out collaboratively by Huan Cui, Wanying Mou, Ning Yu, and Fengrun Sun. Afang Zhu and Lulu Ma revised the article.

## Declaration of interests

The authors declare that they have no competing interests.

## Declaration of generative AI and AI-assisted technologies in the writing process

During the preparation of this work, the authors used ChatGPT (OpenAI) to improve the English language and readability of the text. After using this tool, the authors reviewed and edited the content as needed and take full responsibility for the content of the publication.

## STAR★Methods

### Key resources table

**Table:** 

REAGENT or RESOURCE	SOURCE	IDENTIFIER
**Antibodies**		

C3	Abcam	RRID: AB_2924273
p-NF-κB (Ser536)	Cell signaling technology	RRID: AB_331284
β-actin	Cell signaling technology	RRID: AB_2242334
HRP-conjugated goat anti-mouse	ZSGB-Bio	ZB-5305
HRP-conjugated goat anti-rabbit	ZSGB-Bio	ZB-5301
GluR1	Abcam	RRID: AB_2113447
Syn-1	Synaptic systems	RRID: AB_10805139
C3aR	Santa Cruz	RRID: AB_2066736
GFAP	Abcam	RRID: AB_449329
p-NF-κB (Ser536)	Cell signaling technology	RRID: AB_331284
C3	Abcam	RRID: AB_2924273
Donkey Anti-Rabbit Alexa 488	Abcam	RRID: AB_2636877
Donkey Anti-Rabbit Alexa 594	Invitrogen	A-21207
Donkey Anti-Mouse Alexa 488	Invitrogen	A-21202
Donkey Anti-Mouse Alexa 594	Abcam	RRID: AB_2732073

**Bacterial and virus strains**		

AAV9-CaMKIIα-zsGreen-shC3aR	HanBio (Shanghai, China)	N/A
AAV9-CaMKIIα-zsGreen-Null	HanBio (Shanghai, China)	N/A

**Biological samples**		

Thoracic spinal cord from adult male Sprague-Dawley rats (250–300 g)	This study	N/A
Primary astrocytes isolated from postnatal day 2 (P2) Sprague-Dawley rats	This study (derived from rat cerebral cortices)	N/A
Cerebrospinal fluid (CSF) from adult rats	This study (collected from cisterna magna)	N/A

**Chemicals, peptides, and recombinant proteins**		

Pentobarbital sodium	Sigma-Aldrich	P3761
Cold acetone	Sigma-Aldrich	179973
Dulbecco’s Modified Eagle Medium (DMEM)	Gibco	11965092
Fetal bovine serum (FBS)	Gibco	A5670701
Penicillin/streptomycin	Gibco	15140148
Dibutyryl cAMP	Sigma-Aldrich	D0627
Lipopolysaccharide (LPS)	Sigma-Aldrich	L2630
JSH-23 (NF-κB inhibitor)	Selleck	S7351
C3 protein	Lifespan	LS-G13012
Recombinant PBS vehicle	This study	N/A

**Critical commercial assays**		

ELISA Kit for Complement C3	Elabscience	E-EL-R0250
TRIzol Reagent	Invitrogen	N/A
RT Master Mix	Takara	N/A
SYBR Premix Ex Taq™	Takara	N/A
Membrane Protein Extraction Kit	Beyotime	P0033

**Deposited data**		

Raw and processed data (RNA, Western blot, behavioral tests, imaging data)	This paper	Available from corresponding author upon request

**Experimental models: Cell lines**		

Primary astrocytes (postnatal day 2 rat cerebral cortices)	Isolated in-house, Chinese Academy of Medical Sciences	N/A

**Experimental models: Organisms/strains**		

Sprague-Dawley rats (male, 250–300 g)	Experimental Animal Center of the Chinese Academy of Medical Sciences (Beijing, China)	Approval No. 211–2014 (IACUC)

**Oligonucleotides**		

C3 siRNA	Keygen, Nanjing, China	N/A (sequence listed in Table S1)
C3aR siRNA	Keygen, Nanjing, China	N/A (sequence listed in Table S1)
Negative control siRNA	Keygen, Nanjing, China	N/A (sequence listed in Table S1)

**Software and algorithms**		

GraphPad Prism 9.0	GraphPad Software, Inc., San Diego, CA, USA	https://www.graphpad.com
ImageJ	National Institutes of Health (NIH), Bethesda, MD, USA	https://imagej.nih.gov/ij/
Olympus FluoView (FV1000)	Olympus, Japan	N/A
Tanon 5800 Luminescent Imaging system	Tanon Science & Technology Co., Ltd., Shanghai, China	N/A
BioRender	BioRender, Toronto, Canada	https://biorender.com

**Other**		

Electronic von Frey	IITC Life Science, Woodland Hills, CA, USA	N/A

### Experimental model and study participant details

#### Animals

Adult male wild-type Sprague-Dawley rats (8–10 weeks, 250–300 g) were obtained from the Experimental Animal Center of the Chinese Academy of Medical Sciences (Beijing, China). Animals were housed under standard conditions (23 ± 1°C, 12-h light/dark cycle) with *ad libitum* access to food and water. All rats were randomly assigned to experimental groups. All animal procedures were approved by the Institutional Animal Care and Use Committee of the Chinese Academy of Medical Sciences (Approval No. 211–2014) and were conducted in accordance with ARRIVE guidelines. Only male rats were used to minimize hormonal variability; therefore, potential sex differences were not assessed and should be considered a limitation of this study’s generalizability.

#### Primary astrocyte cultures

Primary astrocytes were isolated from the cerebral cortices of postnatal day 2 wild-type Sprague-Dawley rats. Meninges were removed, and cortices were mechanically dissociated. Cells were cultured in DMEM supplemented with 15% fetal bovine serum (FBS) and 1% penicillin/streptomycin at 37°C in a humidified incubator (5% CO_2_/95% O_2_). When cultures reached 95% confluence, dibutyryl cAMP (0.15 mM) was added to induce differentiation. Microglia and oligodendrocytes were removed by orbital shaking (200 rpm, 1 h). The sex of neonatal pups was not determined, as astrocyte cultures were prepared before sexual differentiation. All procedures involving neonatal rats were approved by the same ethics committee as noted above.

### Method details

#### Modified CPTP model

A modified thoracotomy-induced pain model was established. Rats were anesthetized with pentobarbital sodium (50 mg/kg, intraperitoneally) and intubated with a 16-gauge catheter connected to a small-animal ventilator. Ventilation parameters were adjusted according to body weight. A 3-cm skin incision was made along the right fourth intercostal space, followed by blunt dissection of muscle to expose the intercostal musculature. A 1.5-cm incision was made along the cranial edge of the fifth rib, and the intercostal space was retracted 8 mm for 3 h using a retractor. After surgery, muscle and skin layers were closed with 3-0 chromic and nylon sutures, and mechanical ventilation was continued until spontaneous respiration resumed. Sham-operated rats underwent the same procedure without rib retraction. Extending retraction time to 3 h ensured consistent development of CPTP, reducing the number of animals required.

#### Behavioral tests

- Mechanical hyperalgesia: Mechanical withdrawal threshold (MWT) was measured in the thoracic surgical region using an electronic von Frey device. Rats were acclimated for 15 min before testing. Stimulation was applied until behavioral responses (scratching, trunk rotation >180°, shuddering, vocalization) occurred. Three measurements per rat (≥30 s apart) were averaged.
- Cold allodynia: 0.5 mL of cold acetone was applied to the wound region. Behavioral responses (hind paw scratching, turning toward the site) were recorded within 1 min across three independent trials (>5 min apart).
- All behavioral assessments were conducted by experimenters blinded to treatment allocation.

#### Pharmacological and genetic manipulations

- Intrathecal injections: Performed at the L4–L5 intervertebral space under anesthesia. Correct entry was confirmed by tail flick or paw retraction. Drugs included:
- C3 protein (Lifespan, 0.5–1 μg)
- C3 antibody (Abcam, ab200999, 1–2 μg)
- Isotype IgG (CST, 3900, 2 μg)
- C3 siRNA and C3aR siRNA (Keygen, 0.5 μg/μL)
- LPS (200 ng)
- JSH-23 (10–20 μM)
- Treatments were administered on POD3, POD5, and POD7 for CPTP rats.
- Intraspinal AAV injection: Four weeks before CPTP surgery, rats received bilateral injections of AAV9-CaMKIIα-ZsGreen-shC3aR or control virus (500 nL, 60 nL/min, 0.4 mm depth, HanBio) at T4–T5 under pentobarbital anesthesia and laminectomy.

#### CSF collection and ELISA

CSF was collected from the cisterna magna under pentobarbital anesthesia using a 30-G syringe. Samples were centrifuged at 5000 g for 3 min at 4°C, and supernatants were analyzed for C3 levels using an ELISA kit (Elabscience, E-EL-R0250).

#### RNA extraction and qRT-PCR

Total RNA from dorsal horn tissue or cultured astrocytes was extracted using TRIzol. Reverse transcription was performed with RT Master Mix (Takara), and qRT-PCR was carried out with SYBR Green (Takara) on a Bio-Rad CFX96 system. GAPDH served as an internal control. Primer sequences are provided in Table S1.

#### Western blotting

T4–T6 dorsal horn tissue and cultured astrocytes were homogenized in RIPA buffer with protease/phosphatase inhibitors. Proteins were separated by SDS-PAGE, transferred to PVDF membranes, blocked with 5% BSA, incubated with primary antibodies overnight, and probed with secondary antibodies. Bands were detected with a Tanon 5800 imaging system and quantified using ImageJ.

#### Immunofluorescence

- Tissue staining: Rats were perfused with PBS and 4% paraformaldehyde. T4–T6 spinal cord was cryoprotected in 30% sucrose, sectioned (15 μm), permeabilized, blocked, incubated with primary and fluorescent secondary antibodies, and imaged by Olympus FV1000 confocal microscopy. Three sections per rat were analyzed.
- Cell staining: Cultured astrocytes were fixed, permeabilized, blocked, and incubated with antibodies. Fluorescent signals were quantified using ImageJ.

### Quantification and statistical analysis

All values are presented as mean ± SEM. Normality was assessed using the Shapiro–Wilk test. For two-group comparisons, unpaired Student’s *t* test was used. For multiple-group comparisons, two-way ANOVA followed by Bonferroni post hoc tests was performed. In ANOVA, factors included treatment and time point. Exact sample sizes (n) are provided in figure legends, where n refers to the number of animals or independent replicates. Statistical analyses were conducted using GraphPad Prism 9.0, and *p* < 0.05 was considered statistically significant.

### Additional resources

This study did not generate additional resources such as websites, forums, or clinical trial registrations.
