# Supplementary material for: Astrocyte-to-neuron interaction via NF-κB/C3/C3aR mediates chronic post-thoracotomy pain by modulating neuronal GluR1 in spinal dorsal horn
Source: iScience. 2025 Nov 1;28(12):113917. doi: 10.1016/j.isci.2025.113917 (PMC12664382; doi:10.1016/j.isci.2025.113917)
Supplement: Document S1. Figures S1–S5 and Table S1 [file mmc1.pdf]

iScience, Volume *28*

Supplemental information

Astrocyte-to-neuron interaction via NF-κB/C3/C3aR mediates chronic post-thoracotomy pain by modulating neuronal GluR1 in spinal dorsal horn

Wanying Mou, Ning Yu, Fengrun Sun, Huan Cui, Hanyu Zhang, Yan Cao, Sixuan Jin, Chao Ma, Afang Zhu, Lulu Ma, and Yuguang Huang

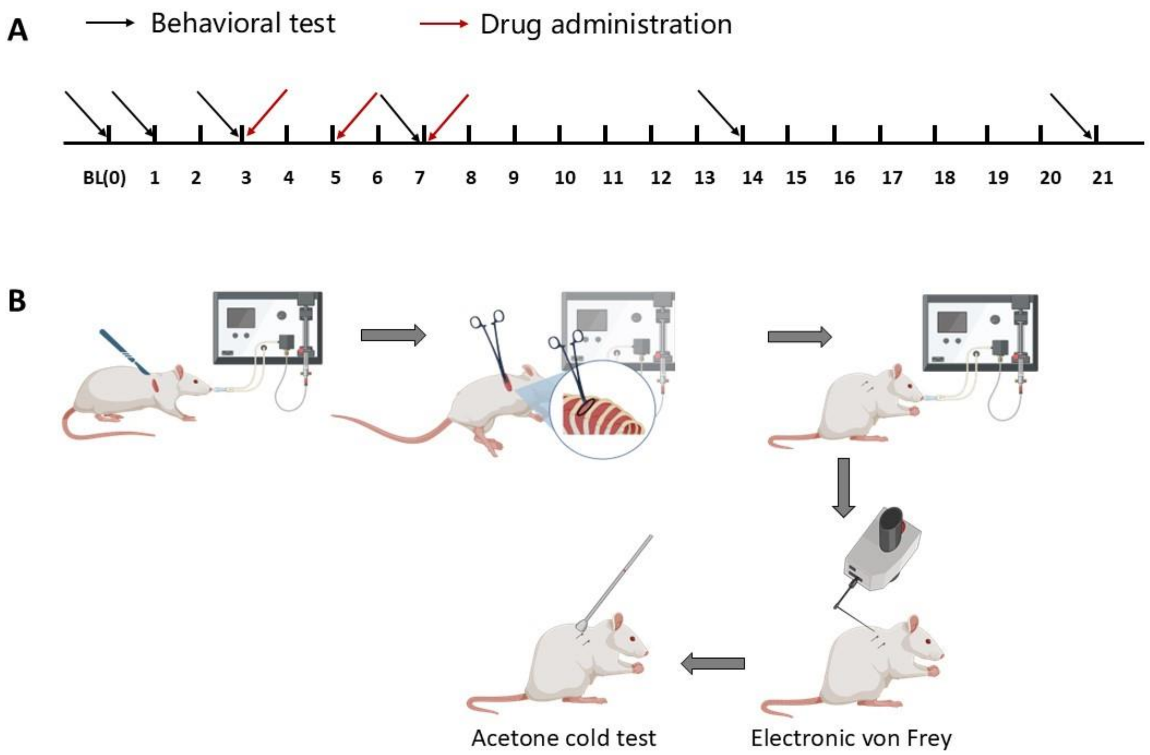

Figure S1. Schematic of the modified chronic post-thoracotomy pain (CPTP) model related to Figure 1.

A. Experimental timeline. Behavioral tests (black arrows) and drug administration (red arrows) were scheduled from baseline (BL) to post-operative day (POD) 21.

B. Diagram of the CPTP model procedures. Thoracotomy was performed under anesthesia, followed by a 3-hour rib retraction to induce persistent postoperative pain. Behavioral assessments, including electronic von Frey and acetone cold testing, were conducted at designated time points.

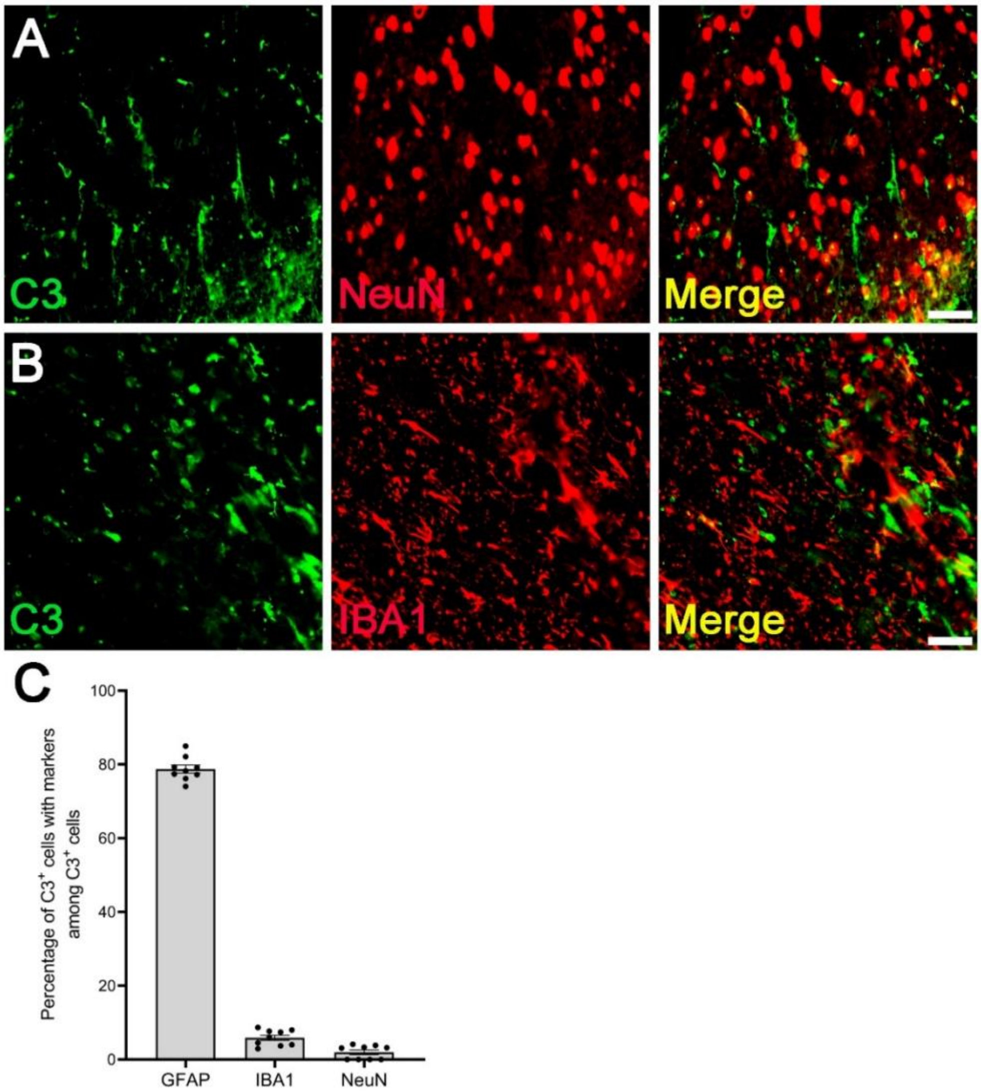

Figure S2. Distribution of C3^{+} cells in the TDH of the CPTP model related to Figure 1.

A. Representative images of TDH stained for C3 (green) and NeuN (red) in TDH.

B. Representative images of TDH stained for C3 (green) and IBA1 (red) in TDH.

C. Percentage of C3^{+} cells with co-stained with markers among all C3^{+} cells.

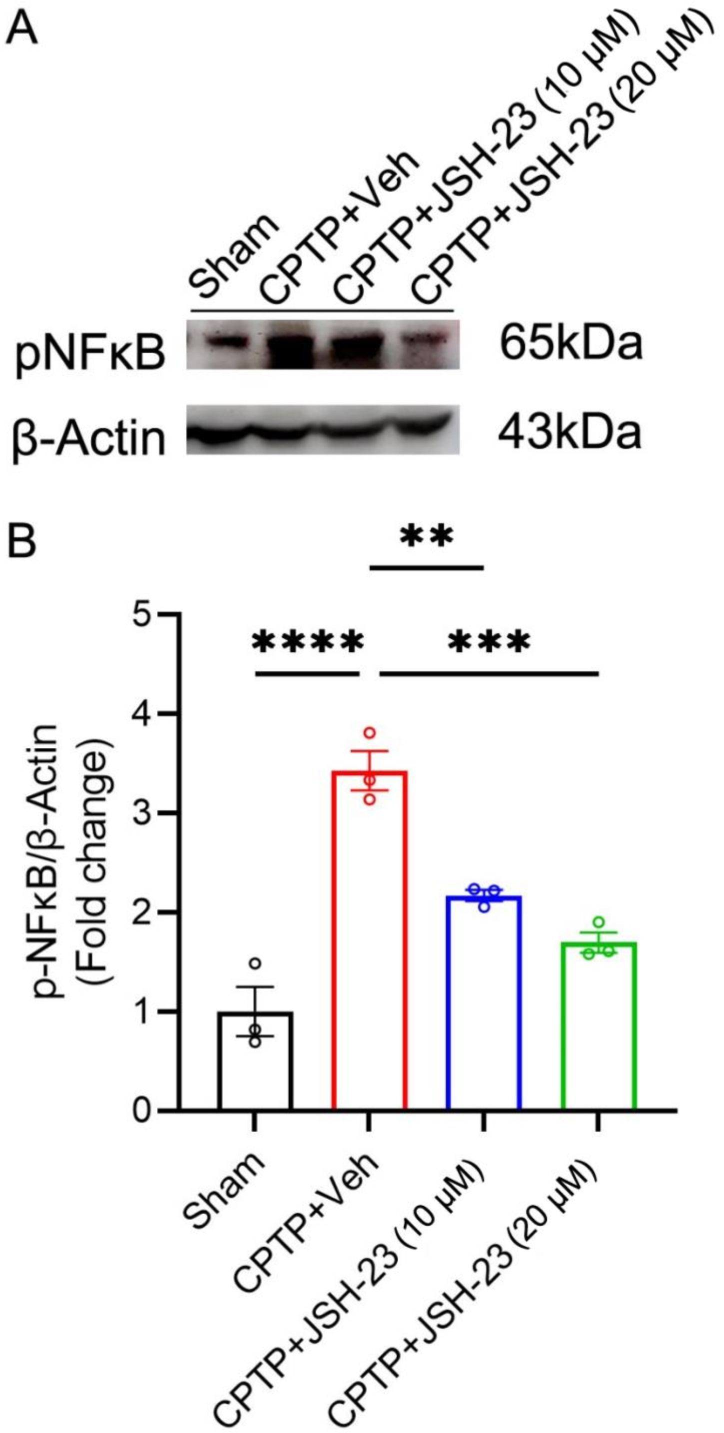

Figure S3. Identification of inhibitory effects of JSH-23 on NF-kb related to Figure 3.

A. Representative images for p-NF-KB and β-Actin among Sham, CPTP+Veh, CPTP+JSH-23 (10uM), and CPTP+ JSH-23 (20 uM).

B. Statistical analysis of relative levels of p-NF-KB. *n* = 3, One-way ANOVA following Bonferroni's post hoc test, ***p<0.01*, ****p<0.001*, *****p<0.0001*.

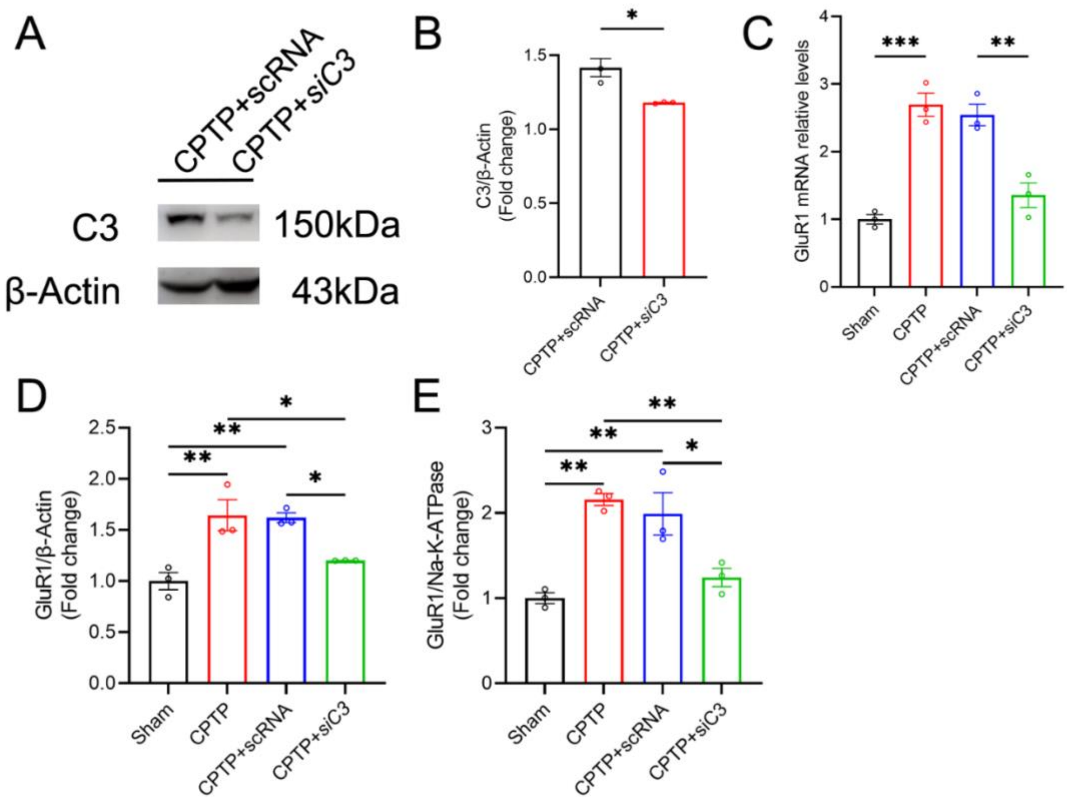

Figure S4. siC3 downregulated GluR1 of TDH in CPTP model related to Figure 5.

A. Representative images for C3 and β-Actin among CPTP+scRNA and CPTP+siC3 groups.

B. Statistical analysis of relative levels of C3. $n = 3$, t test, *$p<0.05$.

C. mRNA expression of GluR1 in the TDH among Sham, CPTP, CPTP+scRNA and CPTP+siC3 groups. $n = 3$, One-way ANOVA following Bonferroni's post hoc test, **$p<0.01$, ***$p<0.001$.

D. Relative expression of cellular total GluR1 in the TDH among Sham, CPTP, CPTP+scRNA and CPTP+siC3 groups. $n = 3$, One-way ANOVA following Bonferroni's post hoc test, *$p<0.05$, **$p<0.01$.

E. Relative expression of membrane GluR1 in the TDH among Sham, CPTP, CPTP+scRNA, and CPTP+siC3 groups. $n = 3$, One-way ANOVA following Bonferroni's post hoc test, *$p<0.05$, **$p<0.01$.

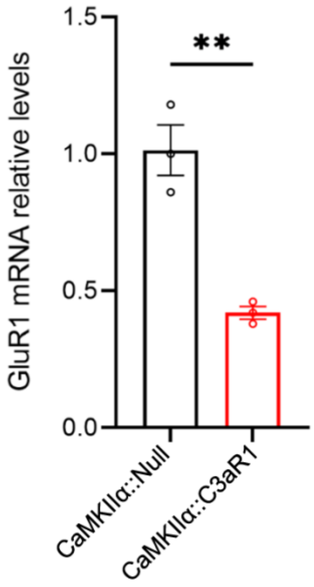

Figure S5. Related to Figure 6.

mRNA expression level of GluR1 in the thoracic dorsal horn (TDH) between the Null and CaMKII::C3aR1 groups. Data are presented as mean ± SEM; *n* = 3 per group. Statistical analysis was performed using an unpaired two-tailed *t*-test. $p < 0.01$.

Table S1. RNA sequences used in this study

Target gene	Primer sequence 5′-3′
C3	Forward: CCTTTCGACCTCATGGTGTTT
	Reverse: CATAGTGCTGTAGGGCTGGG
C3aR	Forward: CTGGAGAAACCTGCCAAGTATG
	Reverse: GGTGGAAGAATGGGAGTTGCT
GAPDH	Forward: CCATGACAACTTTGGCATTG
	Reverse: CCTGCTTCACCACCTTCTTG
